# Exploring Adipose Tissue Behavior in CT: Impact of Age, Sex, and Contrast Media on Body Composition, Liver and Skeletal Muscle

**DOI:** 10.3390/jimaging12070319

**Published:** 2026-07-13

**Authors:** Emil Matthisson, Hanns-Christian Breit, Markus Obmann, Jakob Wasserthal, Martin Segeroth, Daniel Boll

**Affiliations:** 1 Faculty of Medicine, University of Basel, Klingelbergstrasse 61, 4057 Basel, Switzerland; emil.matthisson@stud.unibas.ch; 2 Department of Radiology, University Hospital of Basel, Petersgraben 4, 4031 Basel, Switzerland; hanns-christian.breit@usb.ch (H.-C.B.); jakob.wasserthal@usb.ch (J.W.); daniel.boll@usb.ch (D.B.)

**Keywords:** body composition, computed tomography, visceral adipose tissue, subcutaneous adipose tissue, skeletal muscle, contrast media, image biomarkers, liver attenuation, fatty infiltration

## Abstract

**Objectives:** To evaluate the impact of contrast phase, age, and sex on CT-derived body composition metrics—specifically attenuation and volume of subcutaneous adipose tissue (SAT), visceral adipose tissue (VAT), liver, and skeletal muscle. The potential of the proportion of muscle voxels below 0 Hounsfield units (HU) as a surrogate for fatty infiltration was also explored. **Materials and Methods:** A retrospective analysis of 866 multiphasic abdominal CT scans (non-enhanced [NE], arterial [ART], portal venous [PV]) from 2012 to 2022 was performed. Segmentation of SAT, VAT, liver, and skeletal muscle was conducted using the AI-based TotalSegmentator. Wilcoxon signed-rank tests and Bland–Altman analysis (mean bias and 95% limits of agreement) were applied to assess contrast-related effects; Spearman’s correlation coefficient was used to assess demographic associations. **Results:** Significant variation in attenuation and volume of SAT, VAT, and muscle was observed across contrast phases (*p* < 0.001). SAT attenuation was higher in NE and PV than in ART, while VAT attenuation was highest in PV. SAT volume increased and VAT volume decreased in contrast-enhanced phases. Attenuation and volume showed strong inter-phase correlation (ρ > 0.9). VAT attenuation was significantly higher in females, whereas VAT volume was significantly greater in males. VAT volume negatively correlated with liver attenuation (ρ = −0.33). Muscle voxels <0 HU were significantly reduced in contrast-enhanced scans. **Conclusions:** Contrast phase, age, and sex significantly influence CT-based body composition parameters. These confounding factors should be considered when using quantitative imaging biomarkers in clinical and research settings.

## 1. Introduction

CT-based imaging is fundamental to modern medicine, with an ever-increasing number of examinations worldwide [1,2]. These imaging procedures generate vast amounts of data, much of which remains unused in clinical decision-making. New AI-based methods open up opportunities to make this data usable, for example by segmenting organs and structures [3].

This provides access to image-based biomarkers that can be used, for example, to evaluate bone density, muscle quantity or organ size in various diseases [4,5,6]. These biomarkers can be used for monitoring therapies or opportunistic screening [7].

Further promising image-based biomarkers include data derived from subcutaneous adipose tissue (SAT) and visceral adipose tissue (VAT). An increase in adipose tissue volume, particularly in VAT, is a risk factor for cardiovascular disease, insulin resistance-associated diseases, and atherosclerosis [8,9]. Dysfunctional SAT can lead to an increase in VAT [10]. Therefore, data on SAT and VAT can serve as risk markers for potentially fatal diseases such as type 2 diabetes, strokes or myocardial infarction. Other potential risk markers in this context are associated liver diseases and sarcopenia [8,11,12,13]. Mean attenuation and volume of segmented muscles, VAT, SAT and liver could serve as simple quantitative parameters.

Understanding the dependence of these variables on different contrast phases, as well as on factors like age and sex, is crucial for their application in disease detection and monitoring. Previous studies have demonstrated correlations between the volume, area, and attenuation of subcutaneous adipose tissue (SAT), visceral adipose tissue (VAT), muscles, and the liver, and revealed significant differences in mass and attenuation across contrast phases when contrast agents are used [14,15,16,17]. However, these studies have certain limitations, such as considering only non-contrast phases, examining specific populations, analyzing only certain vertebra levels, or having small sample sizes. A comprehensive understanding of these confounding factors is crucial to ensure accurate interpretation and clinical application of CT-derived body composition metrics.

The study aims to analyze how parameters derived from segmented skeletal muscles, VAT, SAT, and the liver are influenced by age, sex, and contrast phase. Furthermore, statistical correlations between VAT volume, hepatosteatosis, and surrogate markers for fatty infiltration and the quality of selected skeletal muscles will be investigated.

## 2. Materials and Methods

### 2.1. Ethics

This study adheres to the principles of the Declaration of Helsinki and has been approved by the Ethics Committee of Northwest and Central Switzerland (EKNZ; Project ID: EKNZ BASEC 2023-00446; approval date: 29 March 2023). The requirement to obtain informed consent was waived by the Ethics Committee of Northwest and Central Switzerland due to the retrospective nature of the study and the use of anonymized clinical data.

### 2.2. Study Population

A retrospective analysis was conducted on multiphasic CT examinations of the abdomen, including non-enhanced, arterial, and portal venous phases. These scans, performed at a tertiary care center between January 2012 and December 2022, aimed to detect active abdominal bleeding.

Exclusion criteria included examinations with the presence of free intra-abdominal fluid (intra-abdominal bleeding or ascites), non-enhanced examinations with residual contrast agent from a previous examination, incorrect segmentation, incomplete data sets (missing phases, incomplete coverage of the abdomen, lost slices) and processing errors. Additionally, data sets that contributed to the training of the TotalSegmentator algorithm were omitted from this analysis to avoid bias.

Only subjects in which the examined tissue was covered in all contrast phases were compared.

The data was stored anonymously on the Nora Imaging Platform [18], where the segmentation was also performed.

### 2.3. CT Acquisition

CT imaging was performed utilizing either a Somatom Definition Force (2 × 192 slices) or a Somatom Definition AS+ (128 slices) system (Siemens Healthineers, Erlangen, Germany), adhering to a standard clinical protocol that included three contrast phases (non-enhanced, arterial, and portal venous). The protocol specified a tube voltage of 100 kVp. Ultravist 370 (370 mg I/mL; Bayer AG, Leverkusen, Germany) was administered according to a standardized institutional weight-adapted protocol, ranging from 65 mL (50–55 kg) to 105 mL (91–95 kg) in 5 kg increments.

### 2.4. Segmentation

Standardized segmentation was conducted independently for each contrast phase using TotalSegmentator (v2.4.0), without image registration or propagation of segmentations between phases [3]. The regions of interest (ROI) for VAT, SAT, and skeletal muscles were delineated between the upper margin of the thoracic vertebra 12 (T12) and the lower edge of the lumbar vertebra 5 (L5).

### 2.5. Statistical Analysis

The Shapiro–Wilk test was used to assess the normality of the distribution. The median and interquartile range (IQR) were calculated for every variable. A *p*-value of less than 0.05 was considered statistically significant.

To account for multiple comparisons, *p*-values were adjusted using the Benjamini–Hochberg false discovery rate (FDR) method across all hypothesis tests performed.

Unless otherwise stated, liters were used as the unit of measurement for volume. For attenuation, the average Hounsfield unit (HU) value of the tissue was used. Parameters were calculated automatically by the TotalSegmentator.

All statistical analyses and graphs were generated using R software (version 4.3.1).

For VAT and SAT attenuation and volume, the Wilcoxon signed-rank test was used to assess differences between the non-enhanced (NE) and arterial (ART) phases and between the NE and portal venous (PV) phases.

To quantify systematic inter-phase differences, Bland–Altman analysis was performed for each pairwise comparison (NE vs. ART and NE vs. PV), reporting the mean bias (mean of NE − CE differences) and 95% limits of agreement (LoA; bias ± 1.96 × SD of differences).

The effect of age on SAT and VAT attenuation and volume was also examined using Spearman’s correlation coefficient. The non-linearity of age relationships was additionally assessed using generalized additive models (GAM) with penalized cubic regression splines, comparing spline and linear fits by the Akaike information criterion (AIC).

Sex differences in volume or attenuation were examined using the Wilcoxon Mann–Whitney test.

The relationship between VAT volume and liver attenuation was analyzed using portal venous phase data for VAT volume compared with liver attenuation at different contrast phases. The Spearman correlation coefficient was calculated to evaluate this relationship.

The Wilcoxon signed-rank test was used to determine the presence of statistically significant differences in the percentage of values below zero HU between the different contrast phases.

## 3. Results

**Figure 1:**
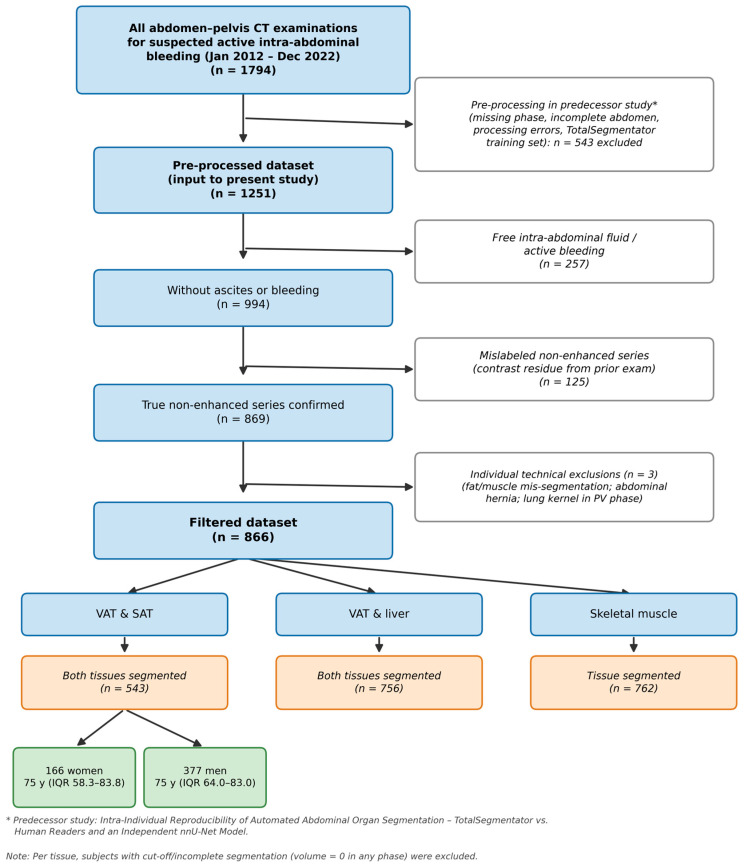
Flowchart outlines the selection of the study population with inclusion and exclusion criteria within the observation window. VAT = visceral adipose tissue; SAT = subcutaneous adipose tissue; IQR = interquartile range. Pre-processing was performed in a predecessor study [19].

From the pre-processed dataset (n = 1251), examinations with free intra-abdominal fluid (active bleeding or ascites) were excluded by a radiologist with three years of experience (n = 257). Mislabeled non-enhanced series containing residual contrast from a prior examination were identified by visual assessment of renal and urinary bladder attenuation, sorted by descending Hounsfield units, and excluded when contrast was present (n = 125). Three further cases were removed for technical errors (fat/muscle mis-segmentation, an abdominal hernia, and a lung reconstruction kernel in the portal venous phase), yielding a filtered dataset of n = 866. Finally, for each tissue, subjects with incomplete (cut-off) segmentation were excluded, resulting in n = 543 for VAT/SAT, n = 756 for VAT/liver, and n = 762 for skeletal muscle (Figure 1 and Supplementary Tables S1 and S2).

**Figure 2:**
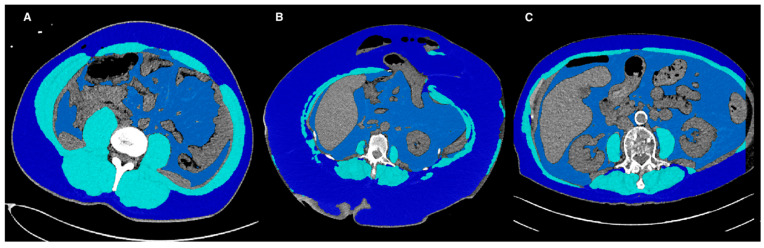
Example CT abdomen slices with TotalSegmentator-derived segmentation. Light blue = skeletal muscle; medium blue = visceral adipose tissue; dark blue = subcutaneous adipose tissue. Examples show (**A**) correct segmentation, (**B**) incorrect segmentation due to an abdominal wall hernia, and (**C**) incomplete segmentations due to incomplete coverage of the left lateral abdomen.

Figure 2 shows examples of correct and incorrect segmentations and incomplete coverage.

Shapiro–Wilk tests indicated that the data were not normally distributed.

### 3.1. Attenuation of Subcutaneous and Visceral Adipose Tissue in Different Contrast Phases

Mean attenuation of SAT in the NE contrast phase (−94.0 HU; IQR, −102.1 to −81.6 HU) was significantly higher compared to the ART contrast phase (−95.5; IQR, −103.0 to −82.2 HU; *p* = 0.001) and significantly lower compared to the PV contrast phase (−86.2 HU; IQR, −95.6–−73.4 HU; *p* < 0.001). There was no significant difference between mean attenuation of VAT in the NE contrast phase (−89.7 HU; IQR, −97.8–−76.2 HU) compared to the ART contrast phase (−89.8 HU; IQR, −97.6–−77.8 HU; *p* = 0.11) and it was significantly lower compared to the PV contrast phase (−79.6 HU; IQR, −89.7–−66.1 HU, *p* < 0.001).

**Figure 3:**
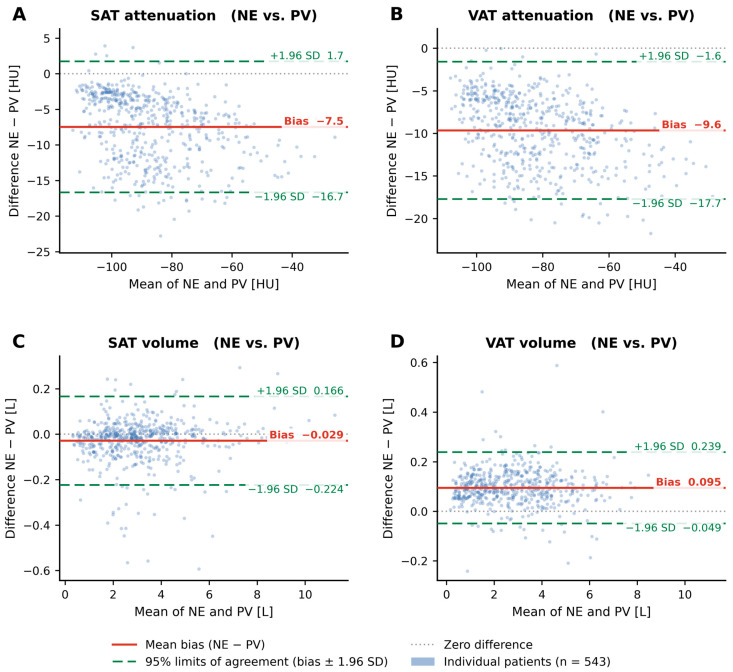
Bland–Altman plots comparing CT-derived body composition parameters between non-enhanced (NE) and portal venous (PV) contrast phases (n = 543). (**A**) SAT attenuation; (**B**) VAT attenuation; (**C**) SAT volume; (**D**) VAT volume. The solid red line indicates the mean bias (NE − PV); dashed green lines indicate the 95% limits of agreement (bias ± 1.96 SD); the dotted gray line marks zero difference. SAT = subcutaneous adipose tissue; VAT = visceral adipose tissue; HU = Hounsfield units; L = liters.

Bland–Altman analysis revealed a small mean bias for SAT attenuation between the NE and ART phases (+1.3 HU; 95% LoA: −9.1 to +11.7 HU), whereas a larger systematic bias was observed between the NE and PV phases (−7.5 HU; 95% LoA: −16.7 to +1.7 HU). For VAT attenuation, the bias between the NE and ART phases was negligible (−0.1 HU; 95% LoA: −10.1 to +10.0 HU), while the NE vs. PV comparison showed a marked systematic shift (−9.6 HU; 95% LoA: −17.7 to −1.6 HU) (Figure 3).

### 3.2. Volume of Subcutaneous and Visceral Adipose Tissue in Different Contrast Phases

SAT volume in the NE phase (2.87 L; IQR, 1.91–3.99 L) was significantly lower (*p* < 0.001) than in the ART phase (2.92 L; IQR, 1.98–4.03 L). Also, the SAT volume of the NE phase compared to the PV phase (2.89 L; IQR, 1.98–4.01 L) was significantly lower (*p* < 0.001). VAT volume in the NE phase (2.64 L; IQR, 1.45–3.96 L) was significantly higher (*p* < 0.001) than in the ART phase (2.58 L; IQR, 1.36–3.86 L). Also, VAT volume in the NE phase in comparison to the PV phase (2.56 L; IQR, 1.35–3.83 L) was significantly higher (*p* < 0.001).

Bland–Altman analysis demonstrated minimal systematic bias for SAT volume between the NE and ART phases (−0.057 L; 95% LoA: −0.26 to +0.15 L) and the NE vs. PV phases (−0.029 L; 95% LoA: −0.22 to +0.17 L). For VAT volume, a small but consistent bias was observed for NE vs. ART ( + 0.076 L; 95% LoA: −0.07 to +0.22 L) and NE vs. PV ( + 0.095 L; 95% LoA: −0.05 to +0.24 L). The limits of agreement for volume were narrower than those for attenuation (Figure 3).

### 3.3. Effect of Age and Sex on Attenuation of Subcutaneous and Visceral Adipose Tissue

**Figure 4:**
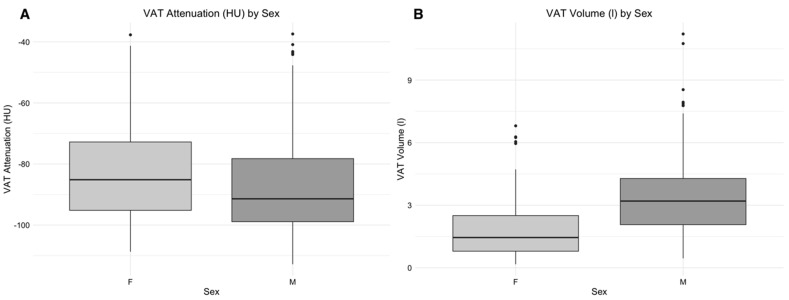
Boxplot of VAT attenuation and volume by sex. (**A**) VAT attenuation was significantly higher in females than in males (*p* < 0.001). (**B**) VAT volume was significantly higher in males than in females (*p* < 0.001). VAT = visceral adipose tissue; F = female; M = male; HU= Hounsfield units; l = liters.

Correlation of SAT attenuation with age in the NE phase was weak but significant (ρ = 0.15, *p* < 0.001, *p* FDR < 0.001, r^2^ = 0.02). There was no significant correlation between VAT attenuation and age in the NE phase (ρ = −0.07, *p* = 0.11, *p* FDR = 0.14). Data for all contrast phases are provided in Supplementary Table S3. Furthermore, the difference in SAT attenuation between male and female patients was not statistically significant in the NE phase (*p* = 0.08). Female patients showed significantly higher VAT attenuation (i.e., less negative HU values) than male patients (−85.2 HU; IQR, −95.2–−72.8 HU vs. −91.4 HU; IQR, −98.9–−78.2 HU, *p* < 0.001, Cohen’s d = 0.29, *p* FDR = 0.001) (Figure 4). Data for all contrast phases are provided in Supplementary Table S4.

### 3.4. Effect of Age and Sex on Volume of Subcutaneous and Visceral Adipose Tissue

There was a significant weak correlation between age and volume for VAT in different phases. These findings could not be observed for SAT. Details are given in Table S5.

There was a significantly higher VAT volume in males (3.20 L; IQR, 2.07–4.28 L) than in females (1.45 L; IQR, 0.80–2.50 L; *p* < 0.001) (Figure 4). Data for all phases are provided in Supplementary Table S6.

Stratified analysis by age group ( < 60, 60–75, >75 years) indicated a non-linear relationship: VAT volume increased with age in younger patients ( < 60: ρ = +0.26; 60–75: ρ = +0.23) but was absent in patients older than 75 years (ρ = −0.03, *p* = 0.65). This non-linearity was confirmed by generalized additive models (GAM) with penalized splines: for VAT volume and SAT attenuation, the spline model fit the age relationship better than a linear model (ΔAIC = +4.4 and +26.1, respectively). Spline fits are shown in Supplementary Figure S1.

### 3.5. Correlation of Liver Attenuation and Visceral Adipose Tissue Volume

The mean attenuation of the liver in the NE phase was 47.6 HU (IQR = 40.8–54.4 HU) and the median VAT volume in the PV phase was 2.55 L (IQR = 1.39–3.85 L). The correlation between liver attenuation and VAT volume was moderate and significant (ρ = −0.33, r^2^ = 0.11, explaining 10.6% of variance; *p* FDR < 0.001).

### 3.6. Effect of Contrast Agent on Percentage of Voxels Below Zero HU, Attenuation and Volume of Skeletal Muscle

**Figure 5:**
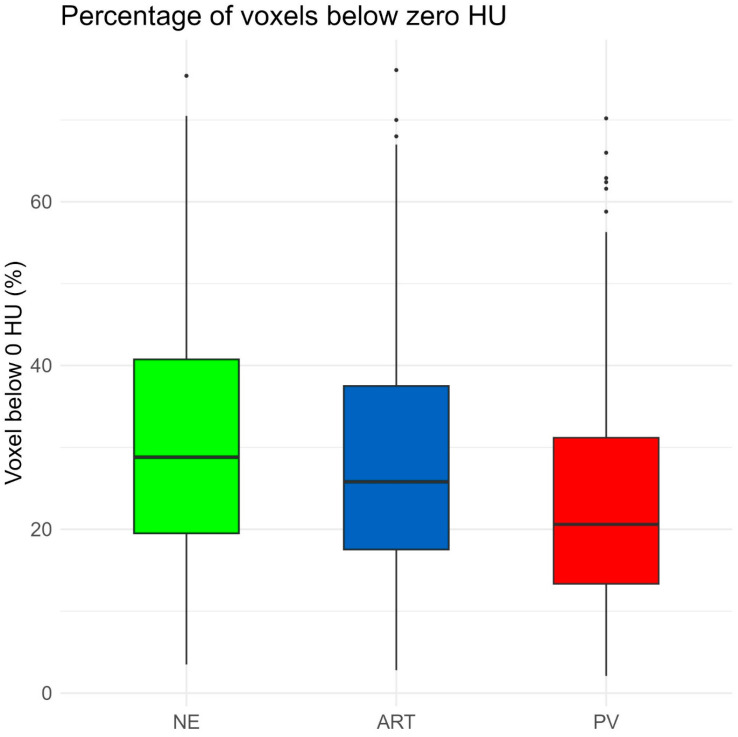
Boxplot of the percentage of voxels below zero HU in the NE phase (green), ART phase (blue) and PV phase (red). Values were significantly (*p* < 0.001) higher in the NE phase compared to the ART and PV phases. NE = non-enhanced; ART = arterial; PV = portal venous.

The percentage of voxels below zero HU in the NE phase (28.8%; IQR = 19.5–40.8%) was significantly higher (*p* < 0.001) than the ART phase (25.8%; IQR = 17.5–37.5%). Also, the NE phase, in comparison to the PV phase (20.6%; IQR = 13.4–31.2%), showed a significantly higher percentage of voxels below zero HU (NE→ART: −2.4 percentage points, −8.3%, Cohen’s d = 1.25; NE→PV: −7.0 percentage points, −26.4%, Cohen’s d = 2.60; both *p* FDR < 0.001) (Figure 5).

Table 1 and Table 2 show that attenuation and the volume of skeletal muscle, respectively, differ significantly in different phases. Skeletal muscle attenuation increased across phases (NE→ART: +2.3 HU, +16.2%, Cohen’s d = 1.26; NE→PV: +8.6 HU, +54.6%, Cohen’s d = 2.88; both *p* FDR < 0.001), whereas muscle volume changed only slightly (NE→ART: +0.7%, Cohen’s d = 0.25; NE→PV: +0.2%, Cohen’s d = 0.07).

## 4. Discussion

The aim of this study was to evaluate the mean attenuation of adipose tissue, liver and skeletal muscles depending on contrast phase, age and sex. Additionally, skeletal muscle was used to test whether the percentage of voxels below 0 HU can be used as a potential surrogate parameter for fatty infiltration depending on the contrast phase.

This is fundamental for further studies because CT-based analysis has been shown to provide valuable regional imaging biomarkers for abdominal body composition assessment and may serve as a complementary or alternative tool to DXA or bioelectrical impedance analysis in selected settings [20]. CT and MRI have emerged as the gold standard for body composition analysis [21], which is utilized for risk stratification in various diseases. Specifically, the measurement of visceral adipose tissue (VAT) is becoming increasingly important as it is a stronger predictor of insulin resistance-related diseases than body mass index (BMI) [10]. In previous studies, adipose tissue was examined at the L4/L5 vertebral segment [22,23,24]. However, there are now some studies that argue for performing body composition analysis at different levels [17,25,26,27,28,29]. It can be concluded from this that there is a lack of consensus among studies on the optimal level at which a tissue should be assessed. One study concluded that single-slice examination should not be used to infer volume from the cross-sectional area of adipose tissue in an individual [30]. It is important to avoid measuring adipose tissue in the thoracic region, as the measurement in the thorax can be significantly influenced by the breath cycle [31]. This is possible using modern AI segmentation algorithms. In addition to volume, it is now possible to determine the mean attenuation of adipose tissue.

Our study shows that there are linear correlations for the mean attenuation in different contrast agent phases for both SAT and VAT. Rather than introducing entirely novel biological findings, this study provides large-scale validation of known confounders affecting CT-derived body composition biomarkers. Its main added value lies in the large multiphasic cohort, fully automated three-dimensional AI-based segmentation, and simultaneous assessment of SAT, VAT, liver, and skeletal muscle within a single analytical framework. For example, Morsbach et al. showed in a group of only 20 patients that the mean attenuation of fat was generally significantly lower in the arterial and portal venous phase than in non-enhanced imaging [32]. Derstine et al. found lower attenuations for visceral adipose tissue in native compared to contrast-enhanced CTs only for cross-sectional-based segmentation in a cohort of 1677 healthy kidney donors [31]. An increase in mean attenuation in the arterial phase compared to the non-enhanced phase for SAT was observed. Gohmann et al. are the only researchers to describe the behavior of three-dimensionally measured VAT and SAT in different contrast phases [14]. These prior studies collectively demonstrate that a contrast phase introduces systematic bias into CT-derived body composition metrics and may require correction or standardization for longitudinal analyses. This is particularly relevant for AI-based automated biomarker extraction, where even small phase-dependent shifts may influence downstream quantitative assessment. Our data generally support the expected increase in attenuation after contrast administration, particularly in the portal venous phase, where both SAT and VAT demonstrated higher attenuation values (i.e., less negative HU values) compared with non-enhanced imaging. The small decrease observed for SAT in the arterial phase was minimal and likely reflects segmentation-related variability or image noise rather than a true biological effect.

Small but statistically significant differences in SAT and VAT volume were observed between contrast phases. These volume shifts are unlikely to reflect true biological changes and can be more plausibly explained by segmentation-related variability. Potential contributing factors include subtle phase-dependent changes in tissue boundary delineation due to contrast enhancement, minor differences in breathing or patient positioning between acquisitions, and variability in AI-based segmentation performance across contrast phases. There was an increase in SAT volume in contrast-enhanced phases and a decrease in VAT volume in contrast-enhanced phases. This is only partly consistent with current studies as, for example, Gohmann et al. observed a significant decrease in VAT, which might be explained by their smaller sample size of 30 patients [14]. This behavior for adipose tissue thus differs from that of the liver, for which no significant difference can be observed between different contrast agent phases, as Winkel et al. have shown [33].

Age and sex had an influence on the mean attenuation and the volume distribution of the VAT. This was not observed for the SAT.

VAT volume and mean attenuation of the liver show a moderate correlation. This observation is consistent with the literature in which VAT is reported as an independent risk factor for hepatic steatosis [34,35].

The number of voxels below 0 HU in a segmented muscle group differs significantly for different contrast agent phases. Previous studies have investigated the significant differences in skeletal muscle attenuation caused by the administration of contrast media [36]. Our study confirms these differences in both attenuation and the percentage of voxels below zero HU. In the context of myosteatosis assessment, this supports the potential relevance of attenuation-based muscle biomarkers but also highlights that the percentage of voxels below 0 HU should currently be considered an exploratory marker due to its sensitivity to contrast phase and segmentation methodology.

Our study has several limitations that need to be discussed: Firstly, this was a retrospective single-center study based on a highly selected tertiary-care hospital population undergoing multiphasic CT for suspected active abdominal bleeding rather than a healthy screening cohort. The extent to which the findings in this patient group correspond to those of the general population must therefore be questioned. On the other hand, a broad prospective study on healthy volunteers is not ethical to realize. Secondly, a specific AI-based segmentation algorithm was used, and all examinations originated from a hospital network using scanners from a single vendor. Therefore, the influence of scanner-specific factors, population characteristics, and segmentation software cannot be fully assessed. In addition, small variations in contrast agent dosage due to weight-adapted administration may have contributed to interindividual variability in attenuation measurements. Furthermore, all contrast phases were segmented independently without image registration. Consequently, small phase-dependent differences, particularly in volumetric measurements, may partly reflect segmentation variability, breathing-related anatomical shifts, or boundary effects rather than true biological changes. Thirdly, further studies are needed to evaluate the value of the measures suggested in this paper for the evaluation of different pathologies, especially in comparison to standard practices. This study only provides fundamental considerations and possible confounders.

## 5. Conclusions

Body composition analysis using CT has great potential due to its broad availability and frequent clinical use. For the variables considered in this work (volume and mean attenuation of SAT and VAT, mean attenuation of the liver, and the fraction of voxels within a muscle group below 0 HU as a surrogate for fatty infiltration), contrast phase, age, and sex represent important confounding factors and should be considered when interpreting quantitative imaging biomarkers. Rather than introducing fundamentally novel biological findings, our results primarily provide large-scale reference data regarding expected phase-, age-, and sex-related variability. These findings may help improve standardization and interpretation of CT-based body composition analyses in future clinical and research applications.

## Figures and Tables

**Table 1:** Comparison of skeletal muscle attenuation between NE and CE.

Contrast	Median NE	IQR NE	Median CE	IQR CE	*p*	*p* FDR
NE vs. ART	15.5 HU	4.9–25.5 HU	18.3 HU	6.9–28.4 HU	<0.001	<0.001
NE vs. PV	15.5 HU	4.9–25.5 HU	24.8 HU	13.7–34.4 HU	<0.001	<0.001

NE = non-enhanced; CE = contrast-enhanced; ART = arterial; PV = portal venous; IQR = interquartile range; *p* = *p*-value of Wilcoxon signed-rank test; *p* FDR = *p*-value after Benjamini–Hochberg false discovery rate correction.

**Table 2:** Comparison of skeletal muscle volume between NE and CE.

Contrast	Median NE	IQR NE	Median CE	IQR CE	*p*	*p* FDR
NE vs. ART	2.12 L	1.67–2.67 L	2.15 L	1.69–2.68 L	<0.001	<0.001
NE vs. PV	2.12 L	1.67–2.67 L	2.14 L	1.69–2.66 L	0.003	0.005

NE = non-enhanced; CE = contrast-enhanced; ART = arterial; PV = portal venous; IQR = interquartile range; *p* = *p*-value of Wilcoxon signed-rank test; *p* FDR = *p*-value after Benjamini–Hochberg false discovery rate correction.

## Data Availability

The data underlying this study are not publicly available due to patient privacy, data protection regulations, and institutional restrictions regarding clinical imaging data. Anonymized aggregated data and analysis scripts may be made available from the corresponding author upon reasonable request and subject to institutional and ethical approval.

## Supplementary Materials

The following supporting information can be downloaded at https://www.mdpi.com/article/10.3390/jimaging12070319/s1.

## Abbreviations

ART	Arterial contrast phase
CT	Computed tomography
DXA	Dual-energy X-ray absorptiometry
HU	Hounsfield units
IQR	Interquartile range
NE	Non-enhanced phase
PV	Portal venous phase
ROI	Region of Interest
SAT	Subcutaneous adipose tissue
VAT	Visceral adipose tissue
