# Supplementary material for: Exploring Adipose Tissue Behavior in CT: Impact of Age, Sex, and Contrast Media on Body Composition, Liver and Skeletal Muscle
Source: J Imaging. 2026 Jul 13;12(7):319. doi: 10.3390/jimaging12070319 (PMC13413376; doi:10.3390/jimaging12070319)
Supplement: Supplementary file 1 [file jimaging-12-00319-s001.zip › jimaging-4370561-supplementary.pdf]

Supplementary Materials

Table S1. Stepwise exclusion cascade and resulting tissue-specific sample sizes.

Exclusion step	n excluded	n remaining
**Pre-processed dataset (input to present study) ***	—	**1251**
Free intra-abdominal fluid (active bleeding or ascites)	257	994
Mislabeled non-enhanced series (residual contrast from prior examination)	125	869
Individual technical errors (fat/muscle mis-segmentation; abdominal hernia; lung kernel in portal venous phase)	3	866
**Filtered dataset**	—	**866**
Tissue-specific: incomplete (cut-off) segmentation, VAT & SAT	323	543
Tissue-specific: incomplete (cut-off) segmentation, VAT & liver	110	756
Tissue-specific: incomplete (cut-off) segmentation, skeletal muscle	104	762

VAT = visceral adipose tissue; SAT = subcutaneous adipose tissue. Cut-off segmentation refers to a tissue volume of zero in any contrast phase, predominantly caused by incomplete coverage of the subcutaneous fat compartment within the CT field of view. The VAT/liver and skeletal-muscle datasets share the common filtered dataset (n = 866) but differ in tissue-specific cut-off exclusions; the VAT & SAT analysis additionally requires complete segmentation of both fat compartments.

* Output of the predecessor study [19], in which missing phases, incomplete abdominal coverage, processing errors, and TotalSegmentator training cases were already removed from the original 1794 examinations.

Table S2. Comparison of demographic characteristics between included and excluded cases.

Characteristic	Included (n = 543)	Excluded (n = 708)
Age, years — median (IQR)	75 (63–83)	76 (63–85)
Sex — male, n (%)	377 (69.4%)	374 (52.8%)
Sex — female, n (%)	166 (30.6%)	334 (47.2%)

IQR = interquartile range (Q1–Q3). Age did not differ significantly between included and excluded cases (Mann–Whitney U test, $p = 0.33$). The sex distribution differed significantly (chi-square test, $p < 0.001$): excluded cases were more frequently female, primarily because incomplete subcutaneous fat segmentation (field-of-view cut-off) occurred more often in obese and female patients (56.0% vs. 30.6% female among SAT cut-off exclusions; $p < 0.001$).

Table S3. Values for the correlation between age and attenuation of SAT and VAT in different contrast agent phases.

Tissue	Contrast phase	Spearman	p Value	p FDR
SAT	NE	0.151	<0.001	<0.001
SAT	ART	0.171	<0.001	<0.001
SAT	PV	0.105	0.014	0.021
VAT	NE	−0.069	0.11	0.143
VAT	ART	−0.042	0.32	0.396
VAT	PV	−0.068	0.11	0.143

Spearman = Spearman's rank correlation coefficient; p Value = p value of Spearman's rank correlation coefficient; p FDR = p value after Benjamini–Hochberg false discovery rate correction; SAT = subcutaneous adipose tissue; VAT = visceral adipose tissue; NE = non-enhanced; ART = arterial; PV = portal venous.

Table S4. Comparison between the attenuation of VAT and SAT in male and female patients.

Tissue	Contrast phase	Median M	IQR M	Median F	IQR F	p Value	p FDR
SAT	NE	−93.6 HU	−101.3 – −81.2 HU	−94.8 HU	−103.8 – −84.1 HU	0.083	0.115
SAT	ART	−94.5 HU	−102.3 – −81.0 HU	−97.0 HU	−104.8 – −83.7 HU	0.048	0.069
SAT	PV	−86.3 HU	−95.3 – −73.5 HU	−85.2 HU	−96.9 – −73.0 HU	0.98	0.984
VAT	NE	−91.4 HU	−98.9 – −78.2 HU	−85.2 HU	−95.2 – −72.8 HU	<0.001	0.001
VAT	ART	−90.9 HU	−98.3 – −78.3 HU	−86.8 HU	−95.3 – −74.5 HU	0.013	0.021
VAT	PV	−81.3 HU	−91.2 – −69.5 HU	−74.4 HU	−85.1 – −62.8 HU	<0.001	<0.001

Median M = median male; IQR M = interquartile range of male (Q1–Q3); Median F = median female; IQR F = interquartile range of female (Q1–Q3); p Value = p value of Mann–Whitney U test; p FDR = p value after Benjamini–Hochberg false discovery rate correction; SAT = subcutaneous adipose tissue; VAT = visceral adipose tissue; NE = non-enhanced; ART = arterial; PV = portal venous. After FDR correction, the difference in SAT attenuation between sexes in the arterial phase was no longer statistically significant (p FDR = 0.069).

Table S5. Correlation between age and volume of SAT and VAT in different contrast phases.

Tissue	Contrast phase	Spearman	p Value	p FDR
SAT	NE	−0.036	0.41	0.479
SAT	ART	−0.032	0.46	0.527
SAT	PV	−0.028	0.51	0.570
VAT	NE	0.227	<0.001	<0.001
VAT	ART	0.230	<0.001	<0.001
VAT	PV	0.225	<0.001	<0.001

Spearman = Spearman's rank correlation coefficient; p Value = p value of Spearman's rank correlation coefficient; p FDR = p value after Benjamini–Hochberg false discovery rate correction; SAT = subcutaneous adipose tissue; VAT = visceral adipose tissue; NE = non-enhanced; ART = arterial; PV = portal venous.

Table S6. Comparison of VAT and SAT volume in different contrast phases depending on sex.

Tissue	Contrast phase	Median M	IQR M	Median F	IQR F	p Value	p FDR
SAT	NE	2.91 L	2.01 – 3.89 L	2.78 L	1.79 – 4.12 L	0.63	0.645
SAT	ART	2.95 L	2.04 – 3.99 L	2.83 L	1.84 – 4.15 L	0.59	0.643
SAT	PV	2.94 L	2.00 – 3.94 L	2.80 L	1.80 – 4.14 L	0.61	0.643
VAT	NE	3.20 L	2.07 – 4.28 L	1.45 L	0.80 – 2.50 L	<0.001	<0.001
VAT	ART	3.15 L	1.99 – 4.27 L	1.37 L	0.71 – 2.42 L	<0.001	<0.001
VAT	PV	3.12 L	1.98 – 4.21 L	1.34 L	0.70 – 2.39 L	<0.001	<0.001

Median M = median male; IQR M = interquartile range of male (Q1–Q3); Median F = median female; IQR F = interquartile range of female (Q1–Q3); p Value = p value of Mann–Whitney U test; p FDR = p value after Benjamini–Hochberg false discovery rate correction; SAT = subcutaneous adipose tissue; VAT = visceral adipose tissue; NE = non-enhanced; ART = arterial; PV = portal venous.

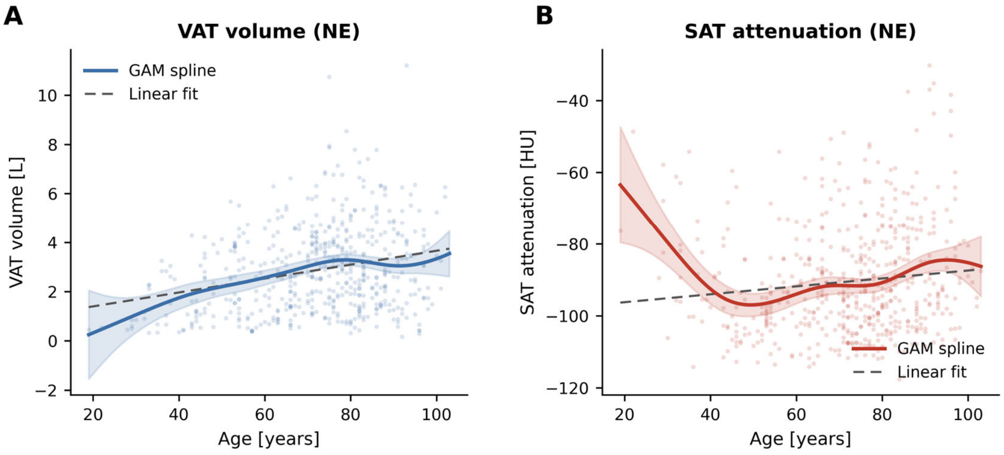

Figure S1. Generalized additive model (GAM) spline fits for the relationship between age and body composition parameters in the non-enhanced phase. (a) VAT volume; (b) SAT attenuation. The colored curve shows the penalized spline fit with its 95% confidence interval (shaded); the dashed gray line shows the corresponding linear fit.

VAT = visceral adipose tissue; SAT = subcutaneous adipose tissue; HU = Hounsfield units; L = liters; GAM = generalized additive model.
